# Enhanced photocatalytic performance of carbon fiber paper supported TiO_2_ under the ultrasonic synergy effect†

**DOI:** 10.1039/d2ra04240a

**Published:** 2022-08-15

**Authors:** Lei Zhang, Jiahui Zhang, Hui Sun, Weiwei Xia, Junhui He, Jie Han

**Affiliations:** School of Physics Science and Technology, Yangzhou University Yangzhou Jiangsu 225002 P.R. China; School of Chemistry and Chemical Engineering, Yangzhou University Yangzhou Jiangsu 225002 P. R. China; National Laboratory of Solid State Microstructures and Department of Physics, Nanjing University Nanjing 210093 P. R. China hsun@yzu.edu.cn

## Abstract

In the present work, TiO_2_ rutile nanorods and anatase nanoflakes have been grown on carbon fiber paper (CFP) by the hydrothermal method. Their photoelectrochemical properties and photocatalytic performances have been investigated. The introduction of CFP is found to improve visible light absorption intensity and effective surface areas apparently, and also make TiO_2_ photocatalysts easier to recycle from aqueous waste. An ultrasonic field was employed during the process of photocatalysis. Sono-photocatalytic efficiency is found to be enhanced significantly in comparison with those of photocatalysis and sonocatalysis, which indicates a positive ultrasonic synergy effect. The scavenger experiments reveal that superoxide radicals (˙O_2_^−^) and hydroxyl (˙OH) are the predominant active species during the dye degradation sono-photocatalytic process assisted by CFP-supported TiO_2_ catalysts. To investigate the ultrasonic synergy photocatalytic effect, the generated amount of reactive oxygen species (ROS) was detected and quantitatively evaluated under visible light, ultrasound, and the combined condition of visible light and ultrasound. As a result, the present work provides an efficient way to improve photocatalytic performance and to realize easy recovery of photocatalyst, which will be helpful for better design of advanced photocatalysts for practical applications.

## Abstract 2

SEM images of TiO_2_(R) nanorods and TiO_2_(A) nanoflakes grown on CFP. And the corresponding catalytic performances under solely visible light, solely ultrasonic field, and the combined conditions of visible light and ultrasonic field.

## Graphical abstract

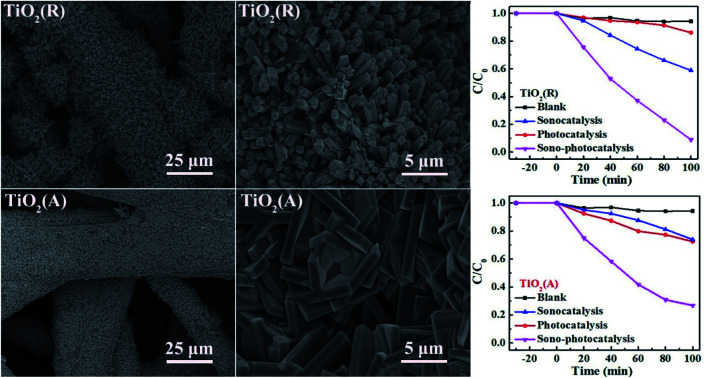

## Introduction

With rapid population growth and industrialization, the consumption of fossil fuels brings about more and more serious environmental contamination.^1–3^ Especially, wastewater contamination is threatening food security, human health, and economic development, so it is urgent to solve these problems with environmentally friendly, low-cost and highly-efficient methods. Photocatalysis can degrade organic pollutants into harmless substances with mild conditions and a simple process and has been considered to be an ideal technology to treat water contamination.^4–7^ At present, titanium dioxide (TiO_2_) is widely accepted as a promising photocatalytic material due to its low cost, high chemical inertness and supreme photocatalytic performance. However, TiO_2_ itself shows poor absorption of visible light and rapid recombination of photoinduced electrons and holes, which results in a lower photocatalytic efficiency.^8–11^ Thus, two solutions were suggested to improve its photocatalytic performance, *i.e*. to expand its spectral response region or to increase its utilization efficiency of photogenerated charges.^12–14^

To construct different types of heterostructures, such as Schottky junctions, p–n junctions, type-II and z-scheme heterostructures *etc*. is generally considered to be the most effective way to improve the separation of photoinduced carriers.^15–18^ Meanwhile, an external field introduced in the process of photocatalysis, such as electric, thermal, magnetic, and ultrasonic field, can greatly favor the photoinduced carrier separation, improve the charge transport, and enhance photocatalytic efficiency. Ultrasonic fields, as a frequently applied noncontact field, is of obvious advantages and is suitable for some nonlaboratory applications.^19,20^ For instance, the joint application of both UV and ultrasound (US) was reported for the photocatalytic reaction of zeolite-loaded MgO nanoparticles (NPs) toward wastewater, the cavitation effect was observed which produces a hot spot phenomenon and accelerates the generation of free and highly reactive radicals that can attack the dye molecules directly.^21^ For the degradation reaction of N-doped TiO_2_ toward antibiotic ciprofloxacin by photocatalysis, sonocatalysis, and sono-photocatalysis, it was found that the simultaneous application of ultrasonic wave and visible light is much more effective than the one used alone.^22^ The microbubble produced by ultrasonic cavitation effect can bring about a violent microflow and shock wave, which will cause a rapid acceleration of the catalyst NPs and improve mass transfer between the liquid phase and the catalyst surface to avoid the accumulation of pollutants and intermediates.^23–28^ All of these factors will favor to improve photocatalytic performance greatly.

Nevertheless, a problem so called secondly contamination of photocatalysts arises in degrading process for aqueous waste, which should not be neglected. As is well known, photocatalysts with the small size and large BET surface area can help to improve the photocatalytic efficiency due to enough surface reactive sites and sufficient activity. Such small-sized particles however are not easy to collect from the wastewater, which causes difficulty for a simple recyclability of the photocatalysts. Fortunately, carbon fiber papers (CFPs) have been introduced as a substrate to grow photocatalysts, which can solve the problem mentioned above.^29–33^ Firstly, CFPs as the catalyst support, can be easily recycled from aqueous waste. Secondly, they can supply a larger surface area for the adsorption of organic reactants and more active sites for photocatalysis. Thirdly, CFPs are conductive, and they may accept photogenerated electrons from TiO_2_, which favors the separation of electron–hole pairs and significantly enhancing photocatalytic performance. Besides, TiO_2_ grown on CFPs could be easily built into photoelectrochemical (PEC) cells, which can keep work in a self-powered mode.^34–37^ However, fewer visible-light self-powered photodetectors with visible-light response has been reported before. It is still a challenge to fabricate an efficient PEC-type TiO_2_ photodetector with larger light response region and surface area to facilitate charge and mass transfer. Finally, CFPs are flexible and stable under corrosive conditions, which are very important in the practical application of catalysts.

In this work, carbon fiber papers were utilized as substrates to grow TiO_2_ nanostructures, so as to enhance visible light absorption. Sine both the crystalline phases and morphologies of TiO_2_ greatly affect the PEC and photocatalytic properties. Therefore, both rutile TiO_2_ nanorods (NRs) and anatase TiO_2_ nanoflakes (NFs) on CFPs were synthesized *via* the hydrothermal route. Their PEC performances were investigated to discuss the transfer rate of carriers, effective active surface area, and energy band structures of both TiO_2_ samples. And their photocatalytic performances were evaluated by degrading process for organic pollutants under visible light irradiation. To improve the photocatalytic efficiency, ultrasonic field was applied in the photocatalytic process, which is so-called sono-photocatalysis and exhibits much more superior photocatalytic efficiency than photocatalysis and sonocatalysis, and their mechanism will be discussed below. The results suggest that ultrasonic synergy is a promising approach for significant improvement in photocatalytic performance and practical application in environmental purification.

## Experimental

### Materials

Commercial carbon fiber papers were purchased from FuelCellStore. Tetrabutyl titanate Ti(OC_4_H_9_)_4_ (≥98%), hydrochloric acid HCl (36 ∼ 38%), and hydrofluoric acid HF (40%) were purchased from Sinopharm Chemical Reagent Company and used without further purification.

### Preparation for rutile and anatase phases TiO_2_ on the CFPs

At first step, tetrabutyl titanate Ti(OC_4_H_9_)_4_ (0.4–0.8 mL) was slowly added into HCl (15–16 mL, 5 M) solution and stirred for 30 min. For preparation of anatase phase TiO_2_, additional 0.3 mL of hydrofluoric acid (HF) was mixed in. The above solution was transferred into the 50 mL teflon-lined stainless-steel autoclave, where CFPs were immersed. The hydrothermal synthesis was conducted at 180 °C for 10–18 h, subsequently the autoclave was cooled to room temperature naturally. The rutile TiO_2_ NRs denoted as TiO_2_(R) was obtained after 18 h heat treatment, and the anatase TiO_2_ NFs denoted as TiO_2_(A) was gained after 10 h heat treatment. Finally, all samples were washed with deionized water and ethanol for three times, and dried at 70 °C for 12 h.

### The evaluation of catalytic activity and reactive oxygen species concentration

The photocatalytic performance of all the samples was evaluated by the photodegrading rhodamine B (RhB) under visible light illumination of a 400 W metal halide lamp. Before irradiation, 50 mg photocatalyst powders were dispersed in 50 mL RhB solution (5 mg L^−1^), and the mixed solution was kept in the darkness and stirred for 30 min to reach an adsorption–desorption equilibrium. In the process of photodegradation, 4 mL suspension was taken out at every 30 min interval, followed by centrifugation to separate the photocatalyst powders. The concentration of the filtrates was determined by measuring the absorption peak intensity at 554 nm with a UV-vis spectrophotometer. The photocatalytic properties of the samples were investigated by photodegrading organic pollutants, such as rhodamine B (RhB: 5 mg L^−1^), Methyl orange (MO: 5 mg L^−1^), and ofloxacin (OFLX: 20 mg L^−1^) aqueous solutions under visible light irradiation of a 400 W metal halide lamp with the wavelength from 380 to 800 nm. The CFP-supported TiO_2_ samples with dimensions of 2 × 1.5 cm^2^ were evaluated by the photocatalysis in 50 mL pollutant solutions. Before photocatalytic reaction, the samples and dye solutions were stirred in the dark for 30 min to achieve the adsorption–desorption equilibrium. During the visible light irradiation, about 4 mL of dye solutions were taken out every several minutes to analyzed their concentrations by UV-2700 UV-visible spectrophotometer. The spectra were scanned and analyzed to determine the concentration of pollutants according to the intensity at 554 nm for RhB, at 460 nm MO, and at 288 nm for OFLX. By putting the photocatalytic reactor into the ultrasonic pool with ∼28 kHz frequency and ∼90 W power, the ultrasonic was applied to promote photocatalytic activities, *i.e*. sono-photocatalysis. The reaction temperature was maintained around 25 °C by adding a small amount of zero-degree water frequently.

The quantity of reactive oxygen species (ROS) generated under the irradiation of ultrasonic only and visible light only, as well as both ultrasonic and visible light was evaluated by the oxidation-extraction photometry (OEP) method. The typical process was described as follows. The TiO_2_ samples with dimensions of 2 × 1.5 cm^2^ firstly were put into 50 mL of 1,5-diphenylcarbohydrazide (DPCI, 1 × 10^−3^ mol L^−1^) solutions to evaluate the production of ROS under visible light, ultrasonic, and both visible light and ultrasonic. Every 20 min, 5 mL of DPCI solution was taken exactly and extracted with mixed solvent of benzene and carbon tetrachloride (volume ratio = 1 : 1), forming diphenylcarbonzone (DPCO) extraction liquids. By using UV-2700 UV-visible spectrophotometer, the concentration of ROS was determined according to the spectra of DPCO extraction liquids whose typical absorption peak at 563 nm. Furthermore, the concentration of ˙OH and ˙O_2_^−^ oxygen radicals was determined by terephthalic acid photoluminescence (TA-PL) and blue tetrazolium (NBT) transformation, respectively. TA can react with ˙OH radicals, and display a specific fluorescence emission maximum at 425 nm. Thus, the amount of ˙OH radicals can be determined by measuring the fluorescence intensity of the peak at 425 nm on a fluorescence spectrophotometer (Hitachi F-4500, Japan). NBT was applied to detect ˙O_2_^−^ due to their reaction production exhibits a maximum absorbance at 259 nm, and the amount of generated ˙O_2_^−^ over catalysts can be measured according to the reduction of NBT on UV-2700 UV-visible spectrophotometer. The quantification experiments of ˙OH and ˙O_2_^−^ were carried out as follows: 2 × 1.5 cm^2^ of CFP-supported TiO_2_ samples was put in 50 mL TA (0.5 × 10^−3^ M) solution or NBT (0.025 × 10^−3^ M) solution, and was exposed to the irradiation of sole visible light, sole ultrasonic, and both visible light and ultrasonic. About 4 mL of the above suspension solution was taken and analyzed every 20 min. The quantification tests of reactive oxygen species were also carried out at 25 °C.

### PEC measurements

The PEC and electrochemical measurements were performed in 0.1 M Na_2_SO_4_ (pH = 7, 25 °C) solution using an electrochemical workstation (CHI 660E, China) with a platinum foil, a saturated Ag/AgCl were served as the counter electrode and reference electrode, respectively. The TiO_2_ nanorods and nanoflakes grown on CFPs with the effective area of 1 × 1 cm^2^ were used as the working electrode and placed in three-electrode cell. A 150 W Xe lamp with 420 nm cut-off filter (CEL-S500) was employed as the visible light source.

### Characterization

The crystal structures of both samples were investigated by an X-ray diffractometer (XRD, XRD-7000, Japan). Field emission scanning electron microscopy (SEM, S-4800II, Japan) was used to observe their morphology. UV-visible absorption spectroscopy was recorded with a UV–Vis–NIR spectrophotometer (Carry 5000, USA).

## Results and discussion

**Fig. 1:**
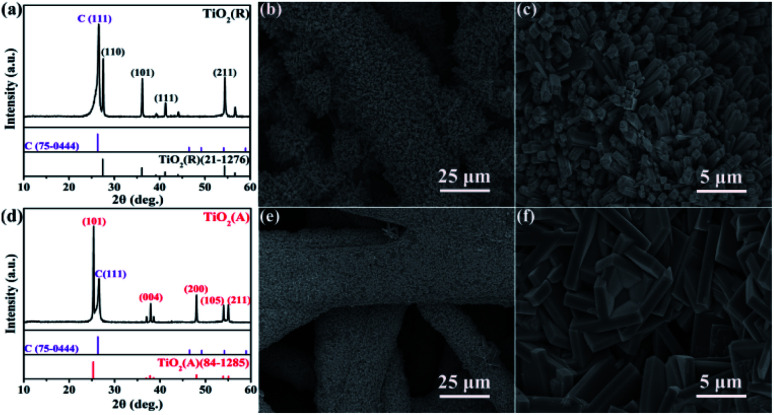
XRD patterns of (a) TiO_2_(R) and (d) TiO_2_(A), and low and high magnification SEM images of TiO_2_(R) nanorods (b and c) and TiO_2_(A) nanoflakes (e and f) grown on CFPs.

Fig. 1 shows the X-ray diffraction (XRD) patterns and SEM images of rutile phase TiO_2_ (TiO_2_(R)) NRs and anatase phase TiO_2_ (TiO_2_(A)) NFs. Here, C (111) diffraction peak can be obviously seen due to the CFP as a substrate. The diffraction peaks of TiO_2_ in rutile (see PDF card no. 21-1276) and anatase (see PDF card no. 84-1285) can be indexed in good agreement. Both the samples are identified to be randomly oriented polycrystalline and single phases without obvious impurities. The morphologies and microstructures for both samples were examined by SEM measurements. The SEM and magnified SEM photographs of in Fig. 1(b) and (c) show the nanorod microstructure of rutile TiO_2_(R), while Fig. 1(e) and (f) display the nanoflake morphology of anatase phase TiO_2_(A). For TiO_2_(R), its nanorod is of approximately 500 nm in diameter and is vertical to the surface of the carbon fiber. For TiO_2_(A), its nanoflake is vertically grown on the carbon fiber as well, with thickness of 600–800 nm and a diameter of 5–8 μm. Both nanorods and nanoflakes are found to be distributed densely and uniformly.

**Fig. 2:**
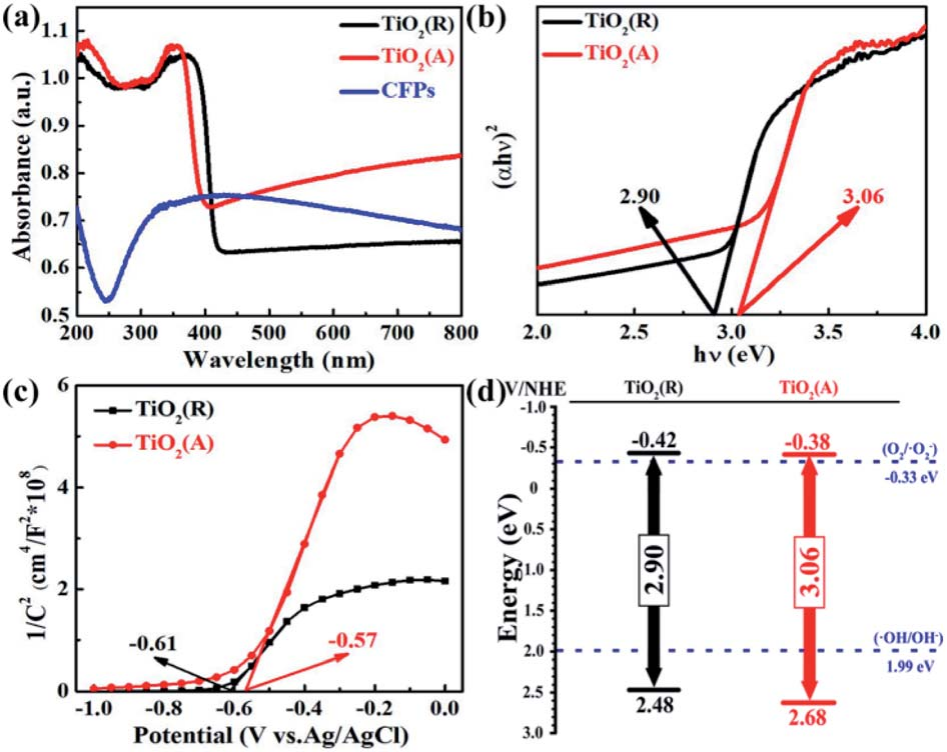
(a) UV-vis spectra, (b) the corresponding plot of (*αhν*)^2^ − *hν*, (c) Mott–Schottky plots, and (d) proposed energy band diagram for both TiO_2_ samples.

Fig. 2(a) suggests UV-vis absorption spectra of both samples with the corresponding plot of (*αhν*) − *hν*^2^ plot shown in Fig. 2(b). And the UV-vis absorption spectra of single CFPs were also given in Fig. 2(a). It is clearly seen that both TiO_2_ samples show intense absorption in the range from UV to visible light, due to the visible light absorption of CFPs.^38,39^ The absorption band edge of TiO_2_ (R) NRs locates at ∼400 nm (Fig. 2(a)), and the corresponding energy gap is evaluated to be ∼2.90 eV (Fig. 2(b)) according to Kubelka–Munk (KM) equation,^40^ whereas the absorption band edge of TiO_2_ (A) NFs is at ∼370 nm (Fig. 2(a)), which indicates the energy gap is ∼3.06 eV (Fig. 2(b)). Therefore, the introduction of CFPs substrate increase visible light absorption apparently and meanwhile brings about a narrow band gap, which favors to promote photocatalytic reaction.

To determine the semiconductor type for both TiO_2_ samples, Mott–Schottky (MS) curves were obtained from the electrochemical impedance spectroscopy measurement as displayed in Fig. 2(c), where *N*_D_ and *N*_A_ denote the concentration of donor and acceptor, and *E*_fb_ refers to flat band potential. The MS equation can be described as:^41^1
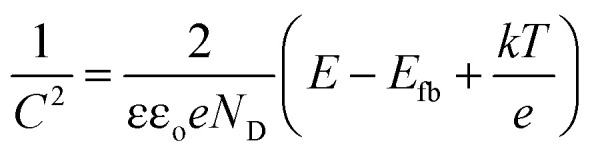
where *ε*_o_ is the vacuum permittivity, *ε* is the relative dielectric constant of TiO_2_, *e* is the electron charge, *k* is the Boltzmann constant, and *T* is the absolute temperature. The positive slopes for the straight-line portions indicate that both of samples are n-type semiconductors in nature. TiO_2_ (R) NRs is of much smaller slope than that of TiO_2_(A) NFs, which implies that the carrier concentration and conductivity of TiO_2_(R) NRs is higher than those of TiO_2_(A) NFs. By extrapolation, the flat band potentials of TiO_2_(R) NRs and TiO_2_(A) NFs are estimated to be −0.61 and −0.57 V *vs.* Ag/AgCl (that is −0.42 and −0.38 V *vs.* NHE), respectively. Therefore, the conduction band (CB) position is estimated to be −0.42 eV for TiO_2_(R) NRs and −0.38 eV for TiO_2_(A) NFs, respectively.^42,43^ Based on the characteristic of flat band potentials and band gaps, the energy band diagrams for both samples are shown in Fig. 2(d). The position for conduction band is found to be favorable for oxygen reduction reaction to generate ˙O_2_^−^ radicals, while the position for valence band is helpful for oxidation process to form ˙OH reactive oxygen species.^44^

**Fig. 3:**
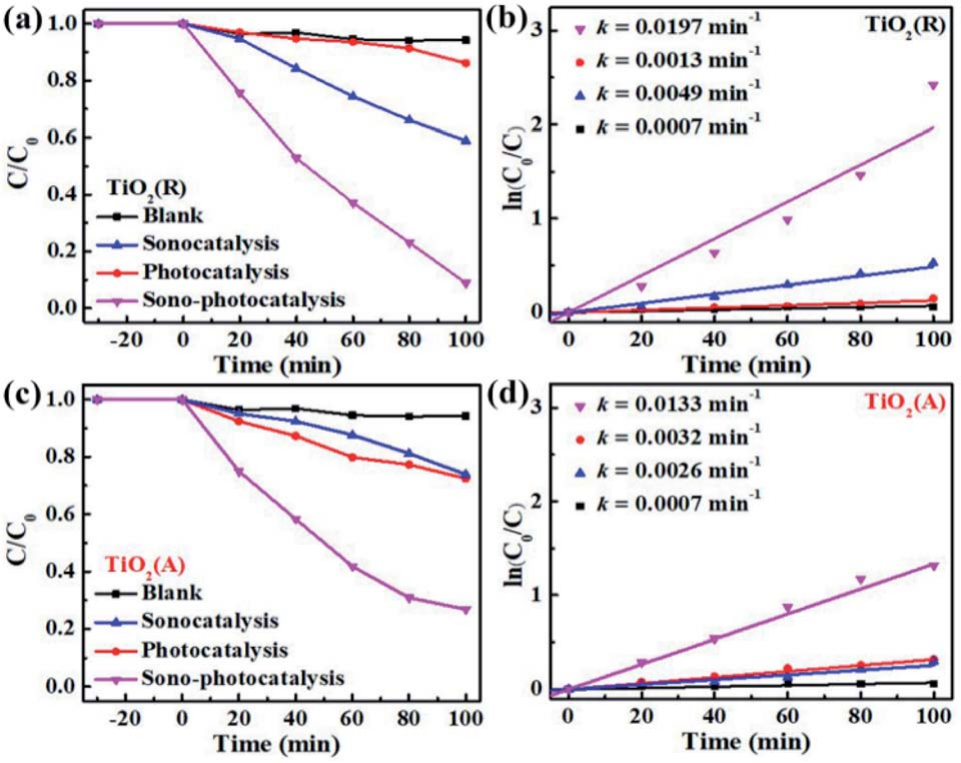
(a and c) RhB degradation over time by TiO_2_(R) NRs and TiO_2_(A) NFs under the irradiation of sole ultrasonic, sole visible light, and both ultrasonic and light. (b and d) Corresponding curves of ln(*C*_0_/*C*) − t.

To evaluate the catalytic performance for both TiO_2_ samples, rhodamine B (RhB) dye degradation experiments were carried out under the irradiation of visible light only (photocatalysis), ultrasonic wave only (sonocatalysis), and both visible light and ultrasonic wave simultaneously (sono-photocatalysis). Fig. 3(a) shows the variation of the concentration of RhB over time under above three conditions. And the corresponding curves of ln(*C*_0_/*C*)-t according to the Lambert–Beer theory is presented in Fig. 3(b). The blank control test without catalyst was performed as well under the irradiation of both light and ultrasonic wave to demonstrate the stability of RhB. In absence of TiO_2_ catalyst, almost no decomposition for RhB is observed, which indicates the self-degradation of RhB is negligible under both light and ultrasonic wave. After illumination of visible light for 100 min, about 27.5% RhB pollutant was found to be degraded by TiO_2_(A) NFs, much larger than that of TiO_2_(R) NRs (13.9%), since TiO_2_(A) NFs exhibits much stronger absorption for visible light than TiO_2_(R) NRs. The photocatalytic performances for both TiO_2_ samples seem to be unsatisfactory. For comparison, the photocatalytic properties for both TiO_2_ phases reported by others are displayed in Table S1.† The catalyst dose of the CFP-supported TiO_2_ with areas of 2 × 1.5 cm^2^ is estimated to be 15 mg, corresponding to the approximate concentration ∼0.3 g L^−1^. The details for photodegradation experiments under ultraviolet light are listed in Table S1.† Above results indicate that the present CFP-supported TiO_2_(A) NFs show better photocatalytic performances.

After 100 min ultrasonic irradiation, the RhB degradation ratios is about 41.2% for TiO_2_(R) NRs and 24.8% for TiO_2_(A) NFs, respectively, which can be likely ascribed to the difference of acoustic cavitation effects. Both TiO_2_ samples are found to exhibit remarkable improvement in catalytic performance and reaction rate *k* under the co-excitation of visible light and ultrasonic. The sono-photocatalytic *k* value of TiO_2_(R) NRs is 15.2 times as large as photocatalytic *k*, and is 4 times as large as sonocatalytic *k* value, which is superior than that reported in Table S1.† Whereas, sono-photocatalytic *k* vale of TiO_2_(A) NFs increases to 3.2 and 4.1 times higher than those of photocatalysis and sonocatalysis, respectively.

**Fig. 4:**
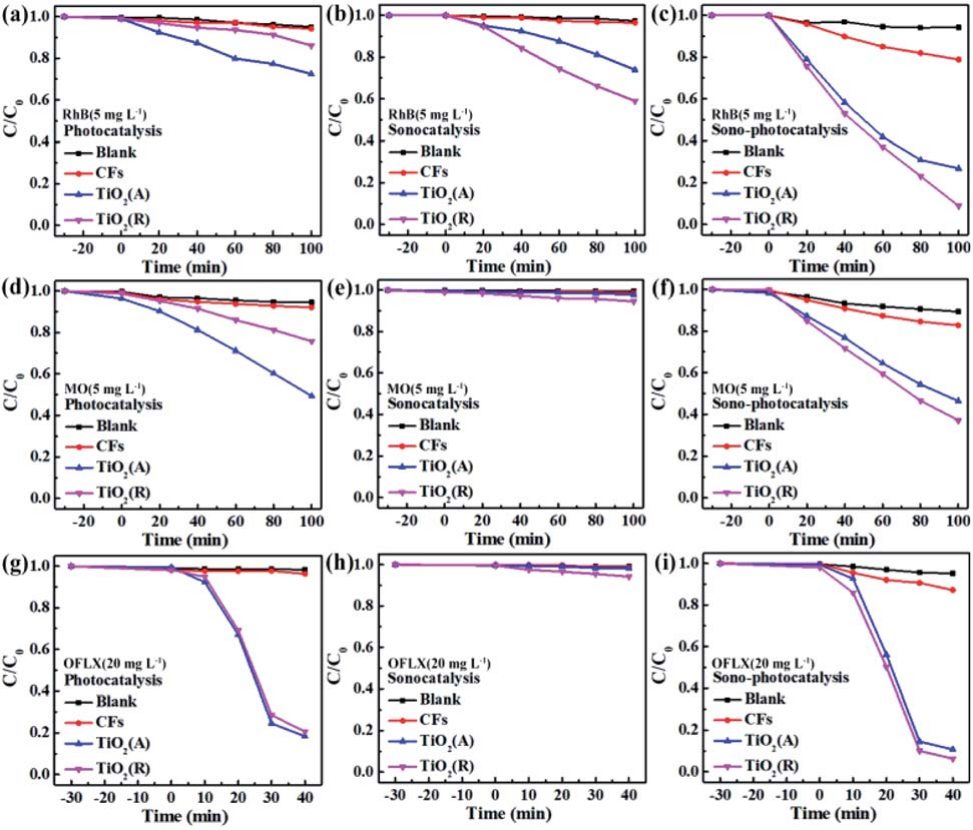
Photocatalytic, sonocatalytic, and sono-photocatalytic degradation for both TiO_2_ samples toward different pollutants.

Both the TiO_2_ samples were further investigated by degrading other pollutants under different external excitations. Anionic methyl orange (MO) dye and antibiotic ofloxacin (OFLX) were chosen as the objective pollutants, as shown in Fig. S1.† The sono-photocatalytic *k* values for TiO_2_(R) NRs and TiO_2_(A) NFs in MO degradation is about 0.0091 min^−1^ and 0.0072 min^−1^, respectively, which are much larger than the corresponding photocatalytic *k* and sonocatalytic *k* value. Similarly, sono-photocatalysis for both samples in OFLX degradation also exhibits the largest reaction rate *k*, in comparison with those of sonocatalysis and photocatalysis. The catalytic performances for both TiO_2_ samples in the degradation of above three pollutants are summarized in Fig. 4. The catalytic efficiency toward pollutant is found to be improved apparently under combined irradiation of visible light and ultrasonic wave as compared to individual illumination. The synergy index (SI) can be quantitatively evaluated by following equation:^20^2
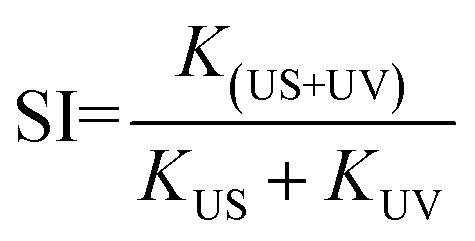
where *k* denotes the catalytic reaction rate constant under different conditions. SI > 1 means a positive effect, while SI < 1 suggests a negative effect.^20^ SI value for both TiO_2_ samples in the sono-photocatalytic process is calculated and listed in Table S2.† The results indicate that synergistic effect toward RhB degradation is more effective than those of MO and OFLX, which needs further investigation in future. It can be concluded that ultrasonic synergy effect plays an important role to improve catalytic performance significantly.

**Fig. 5:**
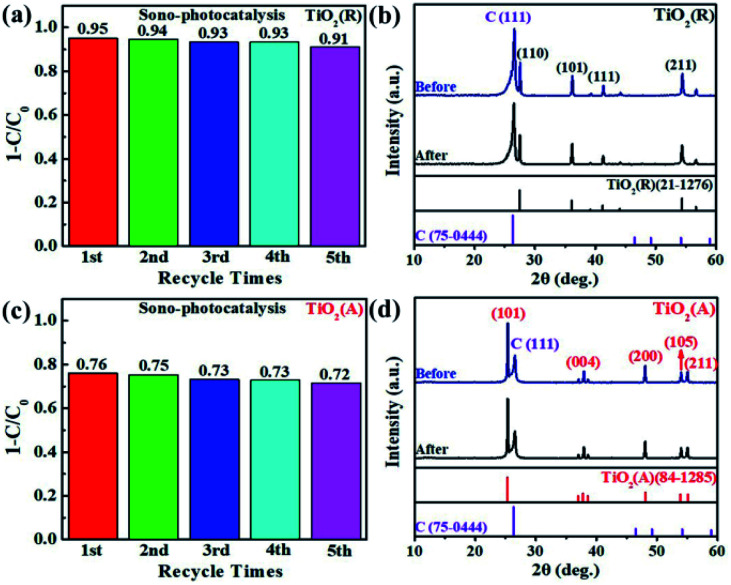
(a and c) Cyclic plots for sono-photocatalytic degradation plots of RhB by TiO_2_ samples, and (b and d) corresponding XRD patterns before and after cyclic experiments.

Cyclic experiments are usually utilized to evaluate the stability of catalysts. Here RhB is chosen as a target pollutant in the cyclic experiments of TiO_2_. Fig. 5 gives the sono-photocatalytic efficiency over cycles and the corresponding XRD patterns of TiO_2_ before and after cyclic experiments. Less than 5% decrease in degradation efficiency is observed and the XRD patterns keep almost same after fifth reaction, which proves that both TiO_2_ samples are of excellent chemical stability.

**Fig. 6:**
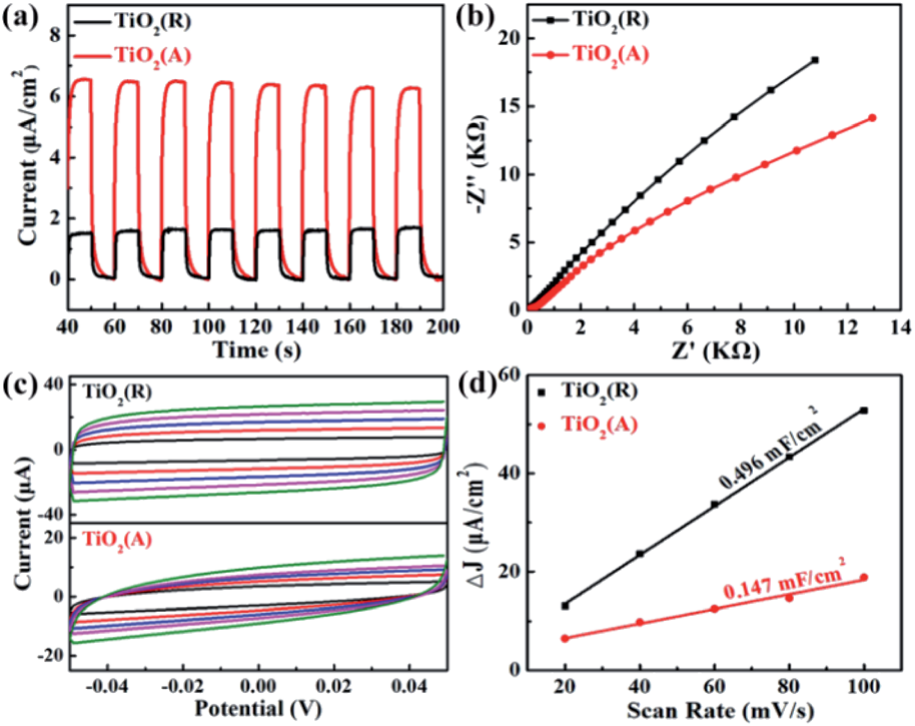
(a) Transient photocurrent responses under on-off 10 s at 0 V *versus* Ag/AgCl, (b) Nyquist plots under dark condition, (c) cyclic voltammetry (CV) at various scan rates, and (d) linear fitting of capacitive current densities *vs.* scan rate for both TiO_2_ samples.

The transfer rate of carriers is known to be closely related with PEC properties. The transient photocurrent measurement for both TiO_2_ PEC detector was carried out using a continuous visible-light pulse with on-off interval of 10 s. The ignorable variation of photocurrent after 10 cycles of on-off illumination switching was observed as shown in Fig. 6(a). The maximal photocurrent of TiO_2_(A) NFs is found to be approximately 6.5 μA cm^−2^, which is nearly 4.1 times as large as ∼1.6 μA cm^−2^ for TiO_2_(R) NRs, which illustrates that TiO_2_(A) NFs has higher photoinduced carrier density and more efficient separation of charge carriers than TiO_2_(R) NRs.^45^ TiO_2_(A) NFs photodetector shows an excellent and reproducible photosensitivity performance, which may improve photocatalytic performance. To investigate the charge transfer and separation at photoanodes, the electrochemical impedance spectroscopy (EIS) has been carried out as presented in Fig. 6(b). The smaller radius of the semicircle at high frequency for TiO_2_(A) NFs indicates the lower charge-transfer resistance, which implies the faster charge transfer and separation.^46^ Due to higher photocurrent density and smaller charge-transfer resistance for TiO_2_(A) NFs exhibits better photocatalytic performance in agreement with the results shown in Fig. 3. Furthermore, to estimate the effective active surface area, cyclic voltammetry (CV) measurement was performed at various scan rate −0.05 ∼ 0.05 V *vs.* Ag/AgCl region, as illustrated in Fig. 6(c). The effective active surface area that is a close correlation with catalytic performance was evaluated by means of electrochemical double layer capacitance (EDLC).^47^ The current densities dependence on scan rate was shown in Fig. 6(d). The EDLC values of TiO_2_(R) NRs and TiO_2_(A) NFs were calculated to be 0.496 and 0.147 mF cm^−2^, respectively, which indicates that TiO_2_(R) NRs possesses much larger active surface area and more active sites than TiO_2_(A) NFs.

**Fig. 7:**
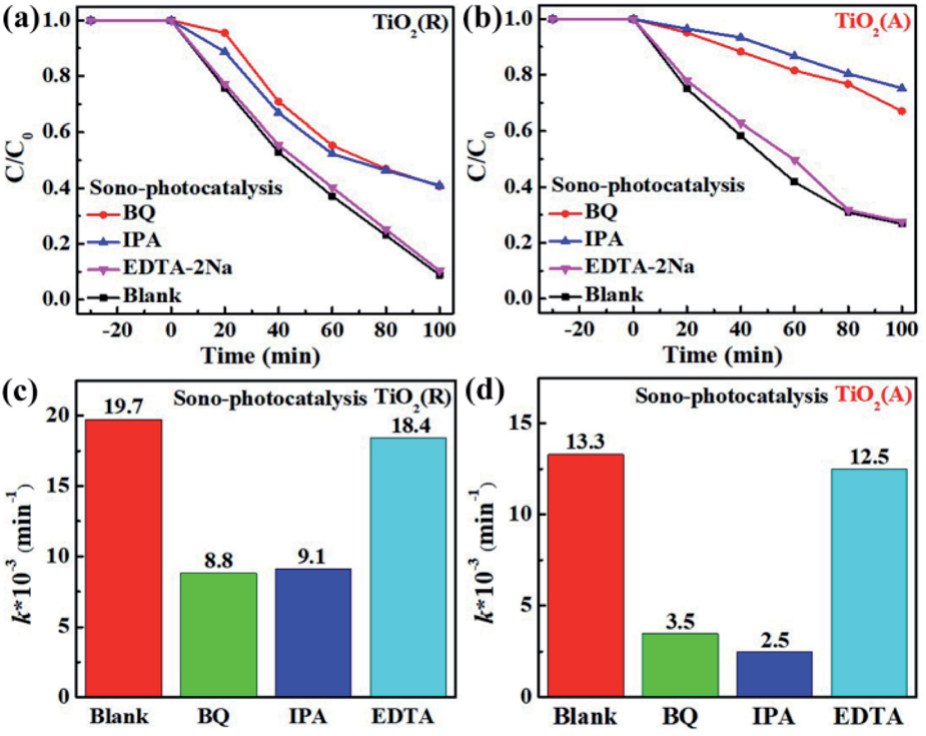
(a and b) The sono-photocatalytic RhB degradation activity with and without scavengers and (c and d) corresponding reaction rate constants *k* by fitting first-order kinetic over TiO_2_(R) and TiO_2_(A) samples.

In order to understand the mechanism of sonocatalytic dynamic process and explore the role of active species in sono-photocatalysis, free radical trapping experiment has been performed. Benzoquinone (BQ), disodium ethylenediaminetetraacetate (EDTA-2Na) and iso-propyl alcohol (IPA) were adopted as scavengers for superoxide radicals (˙O_2_^−^), holes (*h*^+^), and hydroxyl (˙OH), respectively. Fig. 7 displays the sono-photocatalytic degradation of RhB dependent on time with and without the scavengers over both TiO_2_(R) NRs and TiO_2_(A) NFs. After adding EDTA-2Na, the degradation of RhB was almost unchanged, whereas BQ and IPA suppress the degradation efficiency greatly. As shown in Fig. 7(c and d), reaction rate constant *k* decreases rapidly after trapping ˙O_2_^−^ or ˙OH radicals, which implies that ˙O_2_^−^ and ˙OH radicals are two predominant active species during RhB sono-photocatalytic process. The sono-photocatalytic process can be described as following reactions:^24^

3 catalyst + *hν* → e^−^ + *h*^+^

4 *h*^+^ + H_2_O → ˙OH + H^+^

5 O_2_ + e^−^ → ˙O_2_

6 ˙O_2_^−^ + e^−^ + 2H^+^ → H_2_O_2_

7 H_2_O_2_ → ˙OH + ˙OH

8 RhB + ˙O_2_^−^ + OH → intermediates → CO_2_ + H_2_O

As shown above, ˙OH can be formed by reaction (4) and reaction (7).^48^ Suppose that holes h^+^ was the main source for ˙OH, the degradation efficiency then will be significantly inhibited after adding EDTA-2Na as the hole scavenger. On the contrary, trapping holes will seriously slowed down the sono-photocatalytic degradation rate. The capture for ˙O_2_^−^ radicals will dramatically reduce the photocatalytic efficiency as well. It can be concluded that ˙OH is formed by the dominant two-step reaction of ˙O_2_^−^. Holes play little role in dye degradation as well as the production of ˙OH.

**Fig. 8:**
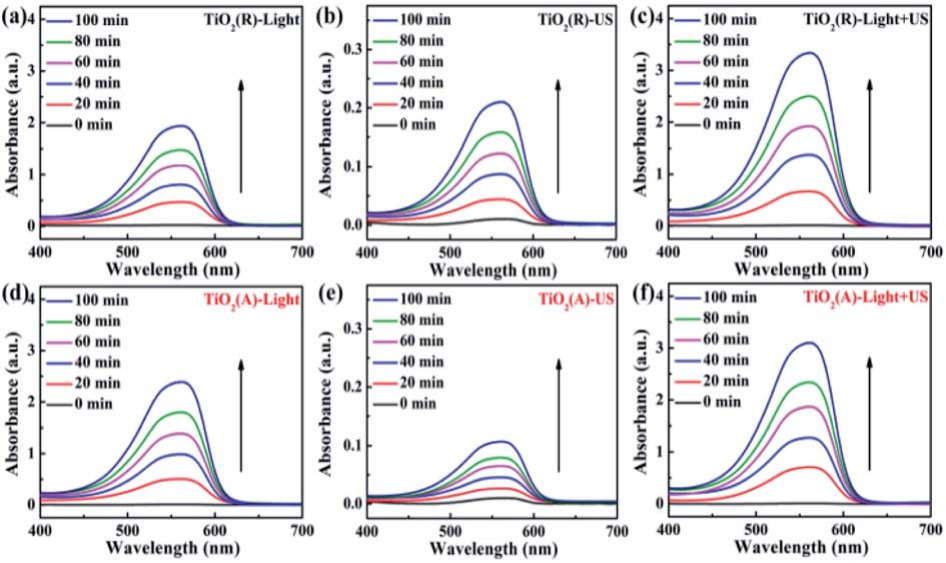
UV-vis spectra of DPCO dependent on time under the different conditions.

To elucidate the synergistic effects for ultrasonic wave in sono-photocatalytic process, the reactive oxygen species (ROS) production experiments were conducted based on oxidation-extraction photometry (OEP) method. In this method, 1,5-diphenylcarbohydrazide (DPCI) can be oxidized by ROS into diphenylcarbonzone (DPCO), and DPCO shows maximum absorption at 563 nm. The ROS quantity can be determined by UV-vis absorption spectrum of DPCO.^25,27,28^Fig. 8 shows the UV-vis absorption spectrum of DPCO extract liquids after 100 min illumination of sole visible light, sole ultrasonic, as well as both light and ultrasonic. For either case, the intensity of DPCO absorption peak increases with the increase of time, suggesting the generated ROS increases with the increasing time, suggesting that the ROS generation increases with time. For both TiO_2_ samples, the intensity for DPCO absorption peak increases significantly under coexisting visible light and ultrasonic wave, compared with the cases under visible light or under ultrasonic wave. For a clear comparison, the UV-vis spectra of DPCO with and without TiO_2_ samples under 100 min illumination of sole visible light, sole ultrasonic, as well as both light and ultrasonic wave are presented in Fig. S2.† Therefore, synergistic effect of ultrasonic wave obviously favors to generate more ROS and to enhance the photocatalytic properties as discussed above.

**Fig. 9:**
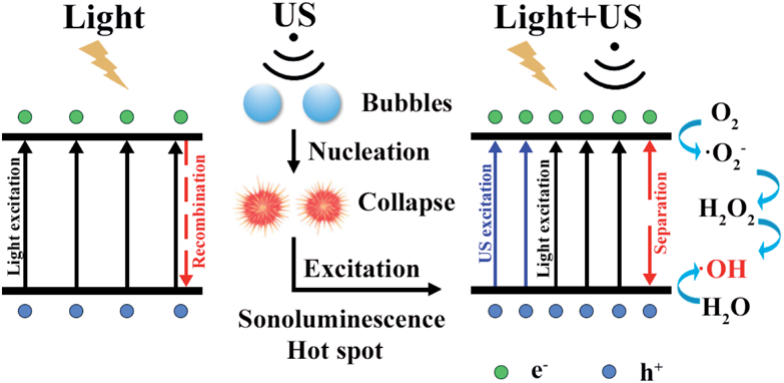
Schematic diagram of TiO_2_ for photocatalytic enhancement under the ultrasonic synergy effect.

There exists obvious difference in ROS experiment for TiO_2_(R) NRs and TiO_2_(A) NFs. TiO_2_(A) NFs can generate more ROS species than TiO_2_(R) NRs under the irradiation of visible light, therefore exhibits the higher photocatalytic efficiency. As discussed above, TiO_2_(A) NFs shows better visible light absorption, higher photogenerated carrier density, and more efficient separation of charge carriers, which will bring about higher photocatalytic efficiency compared with TiO_2_(R) NRs. To clarify the role of ultrasonic wave, the schematic diagram of TiO_2_ photocatalytic enhancement under the ultrasonic synergy effect was depicted in Fig. 9. Seen in the left plot of Fig. 9, electrons stimulated by visible light transfer from valence band (VB) to conduction band (CB), whereas some of them will recombine with holes, which is the reason for inhibited photocatalytic efficiency. Ultrasonic irradiation not only favors to light absorption in a wider wavelength range, but also produces “hot spots” due to cavitation effect, both of which can also promote electrons transition from VB to CB.^24,27,28^ Consequently, under the irradiation of both visible light and ultrasonic wave, more electron–hole pairs will be generated. These electrons and holes can react with the O_2_ dissolved in solution and H_2_O absorbed on the catalyst respectively, to produce ˙O_2_^−^ and ˙OH radicals and to degrade dyes. Therefore, sono-photocatalytic efficiency is much higher than those photocatalysis and sonocatalysis. It was also noticed that TiO_2_(R) NRs is able to generate more ROS radicals under ultrasonic irradiation, which leads to a better sonocatalytic efficiency than TiO_2_(A) NFs. As discussed in PEC properties, TiO_2_(R) NRs possesses much larger effective active surface and more active sites than TiO_2_(A) NFs, which can improve ultrasound function and sonocatalytic activity. For both TiO_2_ samples, the amounts of ROS radicals under combined light and ultrasonic illumination are much greater than the sum of those under individual illumination of light and ultrasonic wave, which may be attributed to so-called synergy effect. In above discussion, a positive synergy effect has been demonstrated by the evaluation for SI value based on the co-contribution of photocatalysis and sonocatalysis. To compare the ultrasonic synergetic effect for different crystal phase TiO_2_ samples, the UV-vis spectra of DPCO after 100 min catalytic reaction under the different condition and corresponding intensity of DPCO peak are presented in Fig. S2.† After 100 min illumination of both visible light and ultrasonic wave, TiO_2_(R) NRs produces 7.4% ROS radicals more than that of TiO_2_(A) NFs, showing stronger sono-photocatalytic ability. In addition to the cavitation effect, ultrasound can promote mass transfer between the liquid phase and the catalyst surface as well, then catalytic reaction is accelerated.^49^ TiO_2_(R) NRs is of more effective surface areas and more active sites for catalytic reaction, therefore exhibiting better sono-photocatalytic performance than TiO_2_(A) NFs.

**Fig. 10:**
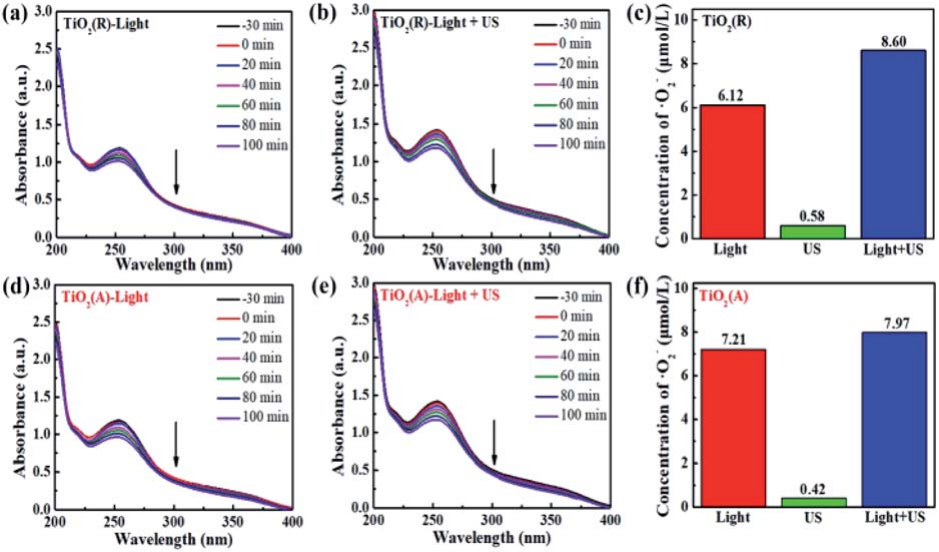
(a–e)The absorbance of NBT molecule at 259 nm under visible light and visible light with ultrasonic wave. (c and f) Bar chart of the concentration of ˙O_2_^−^ over TiO_2_(R) after 100 min under different conditions.

The results for scavenger experiments displayed in Fig. 7 indicate that ˙O_2_^−^ and ˙OH play dominant role in pollutant degradation process. The formation rate of ˙O_2_^−^ and ˙OH species have been investigated to understand the sono-photocatalytic mechanism. NBT and TA-PL transformation are known to be good method to evaluate better the generated amount of ˙O_2_^−^ and ˙OH species, which were performed under the conditions of sole visible light, sole ultrasound, and visible light with ultrasound.^50^Fig. 10(a–e) show the absorption intensity spectra of NBT for both TiO_2_ samples under visible light and visible light with ultrasonic irradiation. The absorption intensity was found to decreases more obviously under case of light plus ultrasonic wave, which means larger ˙O_2_^−^ concentration. To compare the yield of ˙O_2_^−^ under different conditions, the bar chart of the concentration of ˙O_2_^−^ for both TiO_2_ samples are displayed in Fig. 10(c and f). Under illumination of the light plus ultrasonic wave, the ˙O_2_^−^ concentration are evaluated to be 8.60 μmol L^−1^ for TiO_2_(R) NRs and 7.97 μmol L^−1^ for TiO_2_(A) NFs, former is more effective for ˙O_2_^−^ generation.

**Fig. 11:**
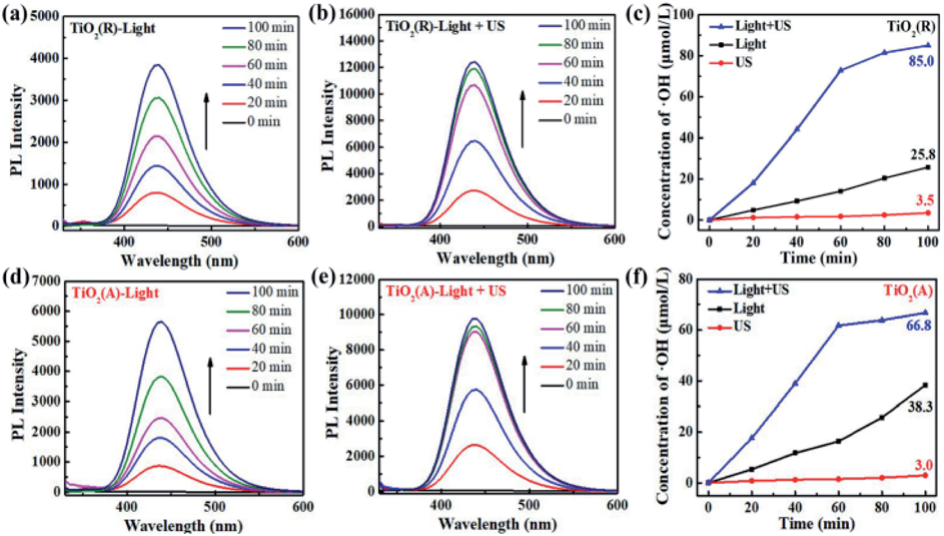
(a–e) Photoluminescence spectra of TA over TiO_2_ under visible light and visible light with ultrasonic wave. (c and f) The variation of ˙OH radical concentration dependent on time under different conditions.

Fig. 11(a–e) show the fluorescence intensity *vs.* wavelength of TA under different condition. The fluorescence intensity of TA is found to increase apparently due to the synergy effect of ultrasound. There exists a relationship between fluorescence intensity and ˙OH concentration can be described as follows:^50^9
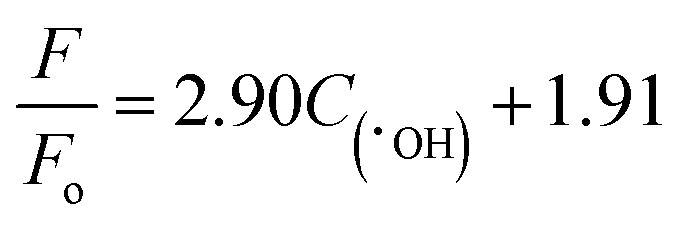
where *F* and *F*_0_ refer to the fluorescence intensity and initial fluorescence intensity of TA solution, respectively. The time dependence of *C*_(˙OH)_ is shown in Fig. 11(c and f). The *C*_(˙OH)_ under both visible light and ultrasound reaches a value ∼85 μmol L^−1^ after 100 min reaction time, which exceeds the sum of the values under sole visible light (∼25.8 μmol L^−1^) and sole ultrasound (∼3.5 μmol L^−1^). As a whole, both the concentration of ˙OH and ˙O_2_^−^ under light plus ultrasonic condition are much larger than the sum of the values under light and under ultrasonic wave, indicating a positive ultrasonic synergy effect.

## Conclusions

Compared with commercial TiO_2_ powders, the TiO_2_ nanostructures rooted on CFPs show obvious advantages. The usage of CFPs can provide large surface areas and ensure enough light absorption and dye molecule adsorption, providing a good conductive channel for carrier transport and improving the photoresponse. The other advantage is the easy recovery as a photocatalyst when degrading aqueous waste. The PEC properties as well as photocatalytic performance for both TiO_2_ samples are investigated to degrade organic pollutants under visible light irradiation were performed. TiO_2_(A) NFs is found to show higher photocatalytic efficiency than TiO_2_(R) NRs, which may be ascribed to its better light absorption. Whereas, TiO_2_(R) NRs is found to exhibit better sonocatalytic performance than TiO_2_(A) NFs, which may be attributed to its larger surface areas. Moreover, the catalytic efficiency is found to be enhanced remarkably under the case of visible light plus ultrasonic wave, in comparison with the case of light or ultrasonic wave, which indicates there exist a positive ultrasonic synergy effect. Such positive synergistic effect of ultrasonic wave may provide an effective way to improve photocatalytic efficiency significantly.

## Conflicts of interest

There are no conflicts to declare.

## Supplementary Material

RA-012-D2RA04240A-s001
