# Supplementary material for: Enhanced photocatalytic performance of carbon fiber paper supported TiO2 under the ultrasonic synergy effect
Source: RSC Adv. 2022 Aug 15;12(35):22922–30. doi: 10.1039/d2ra04240a (PMC9377210; doi:10.1039/d2ra04240a)
Supplement: RA-012-D2RA04240A-s001 [file RA-012-D2RA04240A-s001.pdf]

Electronic Supplementary Material (ESI) for RSC Advances.
This journal is © The Royal Society of Chemistry 2022

Supporting Information for

Enhanced photocatalytic performance of carbon fiber paper supported TiO_2 under the ultrasonic synergy effect

Table S1. Comparison of the catalytic activity of RhB, MO, and OFLX solutions under various conditions by TiO_2 arrays with other reported TiO_2 results.

Dyes	Conditions	Catalyst dose (g/L)	Dye concentration (mg/L)	Time (min)	Degradation (%)	Kinetics rate constant k (*10^{-3} min^{-1})	Reference
RhB	Visible light	0.3	5	100	TiO_2(R)=13.9<br>TiO_2(A)=27.5	TiO_2(R)=1.3<br>TiO_2(A)=3.2	This work
	Ultraviolet light	0.3	5	100	TiO_2(R)=40.1<br>TiO_2(A)=62.2	TiO_2(R)=5.1<br>TiO_2(A)=8.7	This work
	Ultraviolet light	2.5	10	100	TiO_2(A)=27	/	[1]
	Ultraviolet light	1.6	10	100	TiO_2(A)=78	/	[2]
	Ultraviolet light	2.5	6	100	TiO_2(A)=28	/	[3]
	Ultrasonic 90W 40kHz	0.3	5	100	TiO_2(R)=41.2<br>TiO_2(A)=24.8	TiO_2(R)=4.9<br>TiO_2(A)=2.6	This work
	Ultrasonic 100W 40kHz	1.0	10	100	TiO_2(R)=27.8	TiO_2(R)=2.9	[4]
MO	Visible light	0.3	5	100	TiO_2(R)=24.3<br>TiO_2(A)=50.8	TiO_2(R)=2.5<br>TiO_2(A)=6.2	This work
	Ultraviolet light	0.2	3	100	TiO_2(A)=64	/	[5]
	Ultraviolet light	0.25	20	150	TiO_2(A)=78	/	[6]
OFLX	Visible light	0.3	20	40	TiO_2(R)=79.3<br>TiO_2(A)=81.6	TiO_2(R)=27.8<br>TiO_2(A)=30.4	This work
	Ultraviolet light	0.4	20	120	TiO_2(A)=48.6	TiO_2(A)=5.4	[7]
	Ultraviolet light	1.0	10	120	TiO_2(A)=85	TiO_2(A)=71.3	[8]

References

[1] K. R. Reddy, K. V. Karthik, S. B. B. Prasad, S. K. Soni, H. M. Jeong and A. V. Raghu, *Polyhedron*, 2016, **120**, 169-174.

[2] T. S. Natarajan, M. Thomas, K. Natarajan, H. C. Bajaj and R. J. Tayade, *Chem. Eng. J.*, 2011, **169**, 126-134.

[3] H. A. Kiwaan, T. M. Atwee, E. A. Azab and A. A. El-Bindary, *J. Mol. Struct.*, 2020, **1200**, 127115.

[4] J. Wang, Y. Guo, B. Liu, X. Jin, L. Liu, R. Xu, Y. Kong and B. Wang, *Ultrason. Sonochem.*, 2011, **18**, 177-183.

[5] T. A. Saleh and V. K. Gupta, *J. Colloid. Interf. Sci.*, 2012, **371**, 101-106.

[6] L. Lu, R. Shan, Y. Shi, S. Wang and H. Yuan, *Chemosphere*, 2019, **222**, 391-398.

[7] R. Patidar and V. C. Srivastava, *Chem. Eng. J.,* 2021, **403**, 125736.

[8] E. Hapeshi, I. Fotiou and D. Fatta-Kassinos, *Chem. Eng. J.*, 2013, **224**, 96-105.

Table S2. Summary of synergy index of ultrasonic field during catalytic degradation process toward different pollutants.

Synergy index (SI)	RhB	MO	OFLX
TiO_2(R)	3.18	2.94	1.72
TiO_2(A)	2.29	1.13	1.33

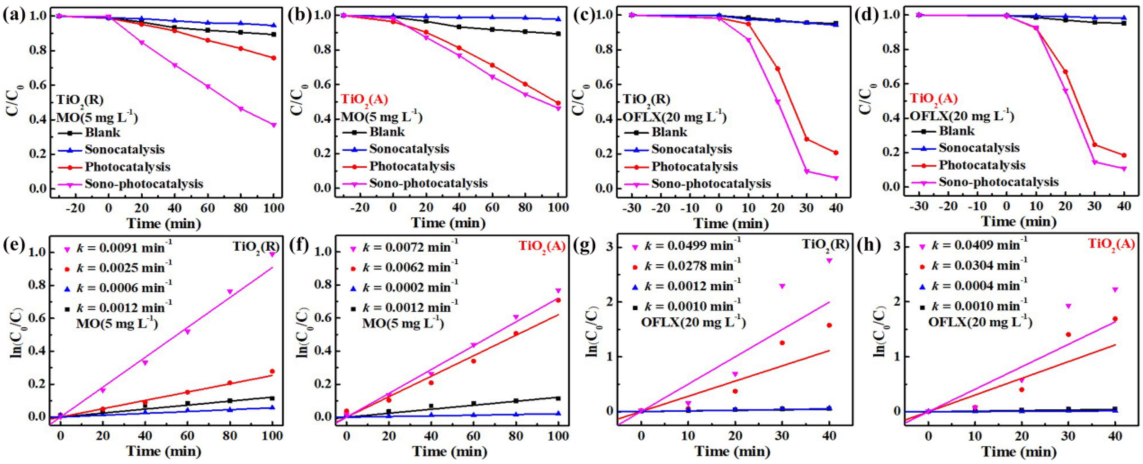

Fig. S1. The degradation efficiency of (a, b) MO and (c, d) OFLX over TiO_2(R) NRs and TiO_2(A) NFs under different conditions. And their corresponding plots of $\ln(C_0/C) - t$

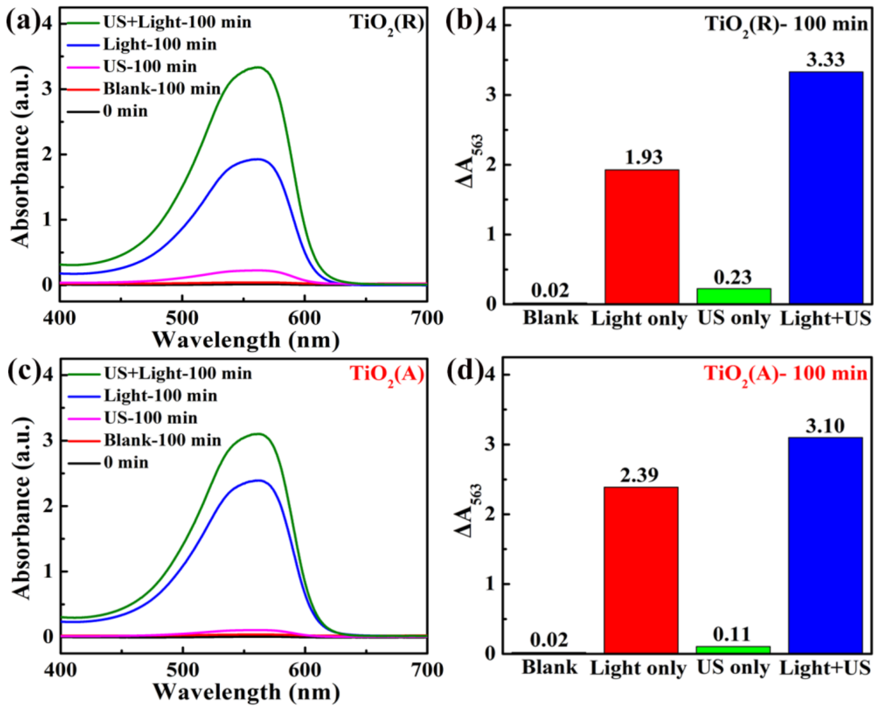

Fig. S2. UV-vis spectra of DPCO under the different condition and (b, d) corresponding intensity of DPCO peak.
